# A Simple Graphical Method to Determine the Order in Catalyst

**DOI:** 10.1002/anie.201508983

**Published:** 2016-01-08

**Authors:** Jordi Burés

**Affiliations:** ^1^ Imperial College LondonDepartment of ChemistryExhibition Road, South KensingtonSW7 2AZLondonUK

**Keywords:** catalysis, kinetic analysis, reaction kinetics, reaction mechanisms

## Abstract

A graphical analysis to elucidate the order in catalyst is presented. This analysis uses a normalized time scale, *t* [cat]_T_^*n*^, to adjust entire reaction profiles constructed with concentration data. The method is fast and simple to perform because it directly uses the concentration data, therefore avoiding the data handling that is usually required to extract rates. Compared to methods that use rates, the normalized time scale analysis requires fewer experiments and minimizes the effects of experimental errors by using information on the entire reaction profile.

Mechanistic studies of catalytic reactions have become more common in academia and industry owing to their value for improving processes and also thanks to the availability of new technology to easily monitor the progress of a reaction. The new reaction monitoring techniques can generate abundant, good‐quality data during the entire course of a reaction, but very few methods have been developed to exploit these features to extract mechanistic information.^1^ Herein, a simple graphical analysis that uses all the reaction profile data to establish the order in catalyst is reported.

The currently available analyses to determine the order in catalyst use rate data. For initial rates, two main analyses are performed: initial rates^2^ against [cat]_T_^*n*^ (T=total) and log–log plots of the initial rates against the analytical concentration of the catalyst^3^ (Figure 1 a). For rates directly measured during the course of a reaction or derived from fitted functions of concentration data, the normalized rate against the concentration of a species is used (Figure 1 b).^4^ Herein, a method to determine the order in catalyst without the need for rate data by directly comparing reaction concentration profiles is described (Figure 1 c).

**Figure 1:**
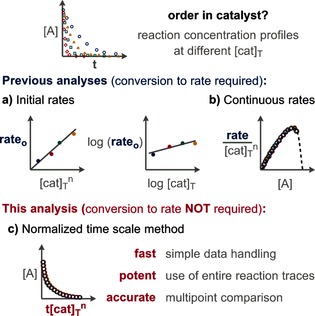
Analyses to determine the order in catalyst.

Although all the analyses hitherto available for determining the order in catalyst use rate data (differential data), few experimental techniques acquire this kind of data directly, and their use is limited because of the intrinsic characteristics of most reactions. There are often two alternative ways to obtain rate data from traces of concentration against time (integral data). The most common one, which is based on initial rates, assumes a linear variation in the concentration of reactants at the beginning of a reaction. This method only uses the data obtained at low conversions or short reaction times,^2^, ^3^ discarding the data from the rest of the reaction and therefore disregarding the associated intrinsic information. The second method fits the concentration data to a preselected function depending on arbitrary parameters, which is further differentiated to mathematically estimate the instantaneous rate at different reaction times.^4^ The arbitrary preselection of a function can bias the results, and the use of a general mathematical function, such as high‐order polynomial functions, can create artifacts in the rate.

The graphical analysis presented in this paper plots the raw (or primary) data of [A] against a normalized time scale, *t* [cat]_T_^*n*^ (Figure 1 c). The adjustment of the time scale for experiments with different catalyst loadings makes the direct comparison of concentration profiles possible. The chosen normalization is theoretically based on the fact that the catalyst concentration is constant during the course of the reaction. Therefore, *t* [cat]_T_^*n*^ becomes one of the parameters of the function that describes the concentration of a reagent at each time point, independently of the complexity of the function. Effectively, the time scale method compresses all traces proportionally to the catalyst loading without altering their shape.^5^

The time normalization is performed by multiplying each time point by the total concentration of catalyst used in each experiment raised to an arbitrary power. This power value should be adjusted until all the corrected conversion curves overlay. This overlay occurs independently of the complexity of the reaction kinetics or changes in the kinetic regime. The graphical interrogation of kinetic parameters has been popularized by Blackmond and co‐workers in the context of the reaction progress kinetic analysis (RPKA).4a, 6 This kind of analysis has gained wide acceptance both in the academic and industrial communities because it is simple to apply and leads to an intuitive interpretation of the results.

Figure 2 a shows how the concentration reaction profiles of a simulated Michaelis–Menten system run with different catalyst loadings change when the scale is normalized for different assumed orders in catalyst. The differences between the normalized concentrations are more pronounced in the late stages of the reaction because the effect of different catalyst loadings in the profiles is accumulative.

**Figure 2:**
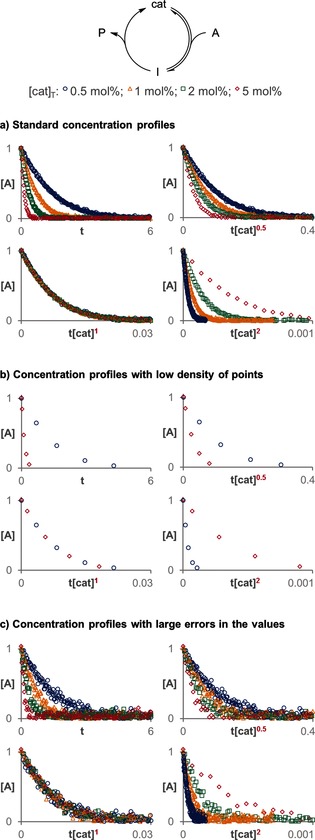
The correct order in catalyst is the one that causes all the curves to overlay (in this case first order).^7^

The normalized time scale method has several advantages over those that involve rate data. The direct comparison of concentration profiles saves time and work because it avoids the previously necessary data handling to extract rate data. The data treatment to extract rates from the raw concentration data can depend greatly on the treatment method. By avoiding this treatment, the data is presented in a more compact way, and the reproducibility is increased.

Moreover, this analysis method requires fewer experiments with different catalyst loadings because it directly compares several points for the entire reaction profile, instead of comparing single points for each reaction, as is the case when using initial rates. Owing to this multipoint comparison, experiments in which only a few data points have been collected, which are intractable with the rate analysis method, can be successfully analyzed. Figure 2 b shows how the order in catalyst can be elucidated with just two traces with four data points each. This characteristic is especially attractive when in situ techniques are not available, and data therefore have to be collected by consecutive sampling or quenching independent reactions. In such cases, it is difficult to collect enough points at short reaction times to extract initial rates or to derive a function with such a low density of points during the entire course of the reaction.

The normalized time scale method is especially beneficial compared to analyses that use rates when the measurement of concentrations contains relatively large errors or there are outliers. Human visual analysis is exceptionally potent in identifying trends that are part of continuous profiles and in minimizing the effect of random experimental errors in single points. Figure 2 c exemplifies this feature by using the same data as in Figure 2 a; however, a random error normally distributed with a standard deviation of 0.05 has been added to the concentration values. Even with such a large error associated with the data points, it is possible to see that the concentration profiles overlay when the time scale is normalized by the total concentration of catalyst raised to the correct power. Conversely, to compensate for the statistical error when initial rates are used to determine the order in catalyst, it would be necessary to use data out of the initial range of concentrations with linear behavior.

Despite all these qualities of the method, there are some caveats that should be taken into consideration. To perform the visual analysis, the normalized abscissa axis should be rescaled to approximately the last value available. The graphs for different orders in catalyst can thus be fairly compared. Owing to the use of visual analysis, there is no mathematical function to describe the error in the determination of the order. Instead, a range of orders leading to a good overlay could be given if necessary. Just as with other analysis methods, it is not possible to determine the order in catalyst if the quantity of catalyst is unknown or if it changes in an unknown way during the reaction. This problem is particularly important when there are fast catalyst deactivation processes that are due to the presence of impurities in comparable concentrations to the analytical concentration of the catalyst.

The normalized time scale method is useful to determine any order in catalyst. Two well‐known catalytic systems have been chosen to illustrate its potency in real cases: the hydrolytic kinetic resolution of terminal epoxides catalyzed by cobalt salen complexes^8^ and the Heck coupling reaction using palladacycle catalysts.^9^

The hydrolytic kinetic resolution of terminal epoxides involves a cooperative mechanism where two discrete catalyst molecules interact in the rate‐determining step of the reaction.^8^ Therefore, the reaction has a second‐order dependency on the catalyst concentration. Figure 3 shows the results that were obtained by applying the normalized time scale method to the corresponding catalytic network, using the kinetic values that had previously been reported.8c Three different traces corresponding to catalyst loadings of 0.85, 0.60, and 0.43 mol % are shown in analogy to the conditions reported in the literature, although two of them would be enough to determine the second order in catalyst.

**Figure 3:**
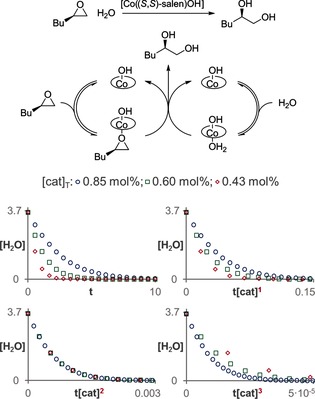
The normalized time scale method shows that the hydrolysis of terminal epoxides is second order in the Co^III^ salen complex.^7^

A more challenging case to determine the order in catalyst is the Heck coupling presented in Figure 4.^9^ The favorable formation of an inactive off‐cycle catalytic dimer that is in fast equilibrium with the corresponding active monomer has been reported. Owing to this equilibrium, the monomer concentration is not linearly proportional to the total concentration of catalyst added, and therefore the order in catalyst depends on the catalyst concentration. The order in catalyst can only vary between first order for very small concentrations of palladium and half order for very high concentrations of palladium.^5^ As shown in Figure 4 a, the theoretical order in catalyst would be around 0.86 for catalyst concentration from 10^−5^ to 2×10^−5^ m and 0.55 for concentrations from 10^−3^ to 2×10^−3^ m. Figure 4 b shows how the normalized time scale method is used to determine the correct order in catalyst, even for these very similar palladium concentrations. The order in catalyst in such mechanistic scenarios is a good indicator of the distribution of the catalyst between monomeric and dimeric species; orders close to one indicate that a major percentage of the catalyst is present as the monomeric species, whereas orders in catalyst close to 0.5 indicate that most of the catalyst is present as the inactive dimer.

**Figure 4:**
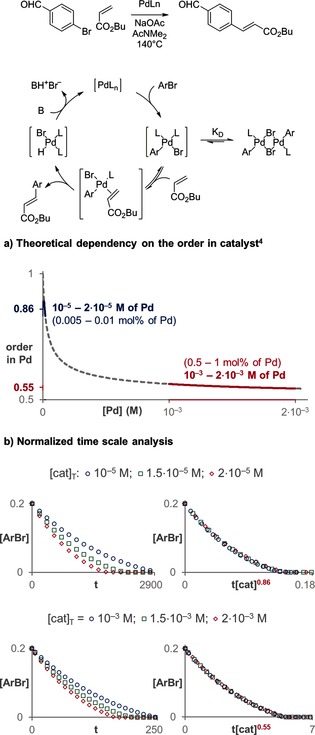
The normalized time scale method enables the differentiation of small changes in the order in catalyst for different concentrations of catalyst.^7^

In conclusion, a practical and powerful method to elucidate the order in catalyst has been presented. The normalized time scale method uses the concentration of a substrate at different time points, thus circumventing the necessity of measuring or deriving rate data. It fully exploits the potential of currently available in situ spectroscopic techniques. This analysis method is simple and fast, it requires fewer experiments than traditional analyses and can even handle sets of data with reduced numbers of points or large random experimental errors. For all of these reasons, the method is expected to attract widespread acceptance and become the preferred option for determining the order in catalyst when using concentration profiles.

## Supporting information

As a service to our authors and readers, this journal provides supporting information supplied by the authors. Such materials are peer reviewed and may be re‐organized for online delivery, but are not copy‐edited or typeset. Technical support issues arising from supporting information (other than missing files) should be addressed to the authors.

Supplementary
