# Supplementary material for: A Simple Graphical Method to Determine the Order in Catalyst
Source: Angew Chem Int Ed Engl. 2016 Jan 8;55(6):2028–31. doi: 10.1002/anie.201508983 (PMC4797368; doi:10.1002/anie.201508983)
Supplement: Supplementary file 1 — Supplementary [file ANIE-55-2028-s001.pdf]

Angewandte Chemie
Eine Zeitschrift der Gesellschaft Deutscher Chemiker

Supporting Information

A Simple Graphical Method to Determine the Order of a Reaction in Catalyst

*Jordi Burés**

anie_201508983_sm_miscellaneous_information.pdf

Supporting Information

Table of Contents:

1. **General Procedures** **S-2**
2. **Integration of the general rate law equation of a catalytic reaction** **S-2**
3. **Adjusted normalized time scale method** **S-2**
4. **Figure 2** **S-3**
5. **Figure 3** **S-4**
6. **Figure 4a** **S-4**
7. **Figure 4b** **S-6**
8. **Numerical data** **S-7**
   - **8.1. Figure 2a** **S-7**
   - **8.2. Figure 2b** **S-12**
   - **8.3. Figure 2c** **S-12**
   - **8.4. Figure 3** **S-17**
   - **8.5. Figure 4b** **S-18**

1. General Procedures

All the figures have been generated by simulating catalytic reactions with a deterministic method LSODA using COPASI and introducing a random error with a normal distribution with Microsoft Excel. The conditions used for the simulation of each catalytic cycle of the manuscript (including the catalytic cycle, the initial concentrations of substances, and the kinetic constant) are detailed below. Also, the resulting exact numerical values used to plot the figures are provided in section "7. Numerical Data" at the end of this supporting information.

2. Integration of the general rate law equation of a catalytic reaction

The normalized time scale method is theoretically based on the fact that catalyst concentration is not a thermodynamic driving force of the reaction and therefore is constant during the reaction. Integration of the general rate law equation of a catalytic reaction shows how $t[\text{cat}]_T^n$ becomes one of the parameters of the function that describes the concentration of a reagent at each time point.

$$-\frac{\mathrm{d[A]}}{\mathrm{dt}} = f([A],[B],\text{kinetic const.})\cdot[\text{cat}]_\mathrm{T}^{\mathrm{n}}$$

$$\int_{[\mathrm{A}]_\mathrm{o}}^{[\mathrm{A}]_\mathrm{t}} f^{-1}([A],[B],\text{kinetic const.})\,\mathrm{d[A]} = \int_0^{\mathrm{t}} -[\text{cat}]_\mathrm{T}^{\mathrm{n}}\,\mathrm{dt}$$

$$F^{-1}([\mathrm{A}]_\mathrm{t},[\mathrm{A}]_\mathrm{o},[\mathrm{B}]_\mathrm{o},\nu_\mathrm{A},\nu_\mathrm{B},\text{kinetic const.}) = -\boldsymbol{t}[\mathbf{cat}]_\mathbf{T}^{\mathbf{n}}$$

$$[A]_t = G\big([\mathrm{A}]_\mathrm{o},[\mathrm{B}]_\mathrm{o},\nu_\mathrm{A},\nu_\mathrm{B},\text{kinetic const.},\boldsymbol{t}[\mathbf{cat}]_\mathbf{T}^{\mathbf{n}}\big)$$

3. Adjusted normalized time scale method

The normalized time scale analysis complements another method for investigating catalyst stability and product inhibition by direct comparison of concentration profiles. Blackmond used a time adjustment to compare reaction profiles of same excess experiments (ref. 1b of the manuscript). The profile curve of the reaction with the lowest initial concentration of reactants has to be shifted by the same amount of time that takes the reaction with the highest initial concentration to achieve the initial concentration of the first one. Both methods, the time adjustment and the time scale, can be simultaneously used to compare same excess experiments with different catalyst loadings. To do so, it is necessary to use the normalized time adjustment, where the standard time adjustment is scaled by the catalyst loading of the reaction with the highest initial concentrations of reactants. The figure below shows the procedure to determine the order in catalyst with two experiments with different catalyst loadings (0.01 M and 0.05 M) and different initial concentration of reactants ([A]: 1.0 M and 0.7 M), but the same excess ([excess] = [B]o – vB/vA [A]o = 0.3 M). The trace for experiment B has to be shifted 0.25 h (the standard time adjustment) multiplied by 0.01 (the total concentration of catalyst for

experiment A) to the power of the order in catalyst. By applying this factor to the time adjustment, the trace starting at the lowest concentration will be shifted to the correct normalized time point. Then the process to find out the order in catalyst is identical to the one previously described.

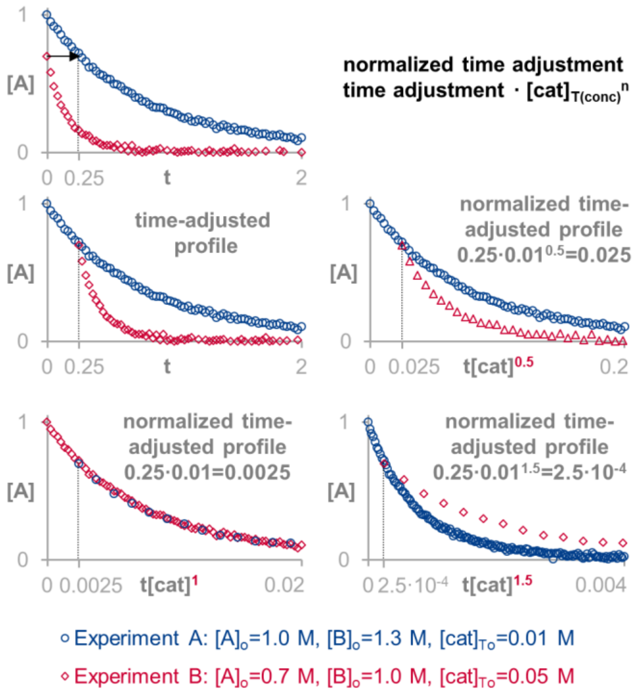

4. Figure 2

The values of concentration and kinetic constants used to simulate figure 2 are shown below.

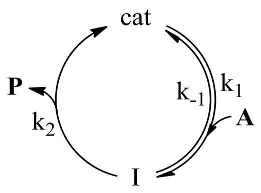

$[A]_o = 1.0$ M;

$k_1 = 0.1\ M^{-1}s^{-1}$; $k_{-1} = 0.1\ s^{-1}$; $k_2 = 0.1\ s^{-1}$

The simulated concentration results were obtained after applying an error with a normal distribution with a standard deviation of 0.01 for figure 2a and 2b and a standard deviation of 0.05 for figure 2c and are listed in sections 7.1 (Figure 2a), 7.2 (Figure 2b) and 7.3 (Figure 2c) of this supporting information.

5. Figure 3

The values of concentrations and kinetic constants used to simulate figure 3 are shown below.

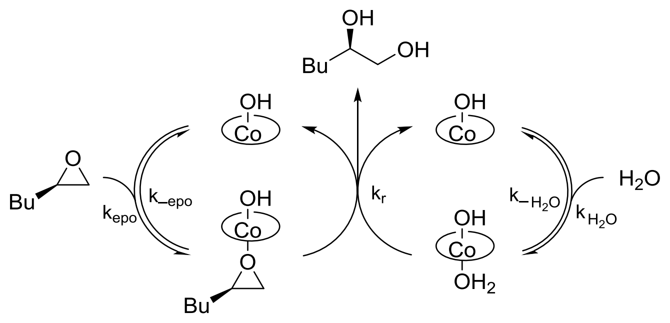

$$[H_2O]_o = 3.63\ M;\ [epoxide]_o = 6.19\ M$$

$$k_{epo} = 10^6\ M^{-1}s^{-1};\ k_{-epo} = 8.4890\cdot10^6\ s^{-1};\ k_{H2O} = 10^6\ M^{-1}s^{-1};\ k_{-H2O} = 5.5556\cdot10^6\ s^{-1};\ k_r = 17.88\ M^{-1}s^{-1}$$

The simulated concentration results against time for the different concentrations of catalyst (0.0308 M, 0.85 mol%; 0.02185 M, 0.60 mol%; 0.0155 M, 0.43 mol%) are in section 7.4 of this supporting information.

6. Figure 4a

The order in catalyst for the catalytic cycle shown in Figure 4 of the manuscript depends on the kinetic constants of the catalytic cycle, the thermodynamic constant of the out-cycle dimerization equilibrium and the concentration of the substrates. The theoretical value of order in catalyst for each set of data is obtained using the normalized sensitivity analysis of the rate law assuming steady-state conditions.

The Bodenstein steady-state approximation is applied to the catalytic cycle and the quasi-equilibrium assumption to the out-cycle equilibrium. The rate law for the reaction (equation 5) is derived using these three equations (equations 1, 2 and 3) and the mass balance (equation 4) for the catalytic species. The notation has been simplified as shown below.

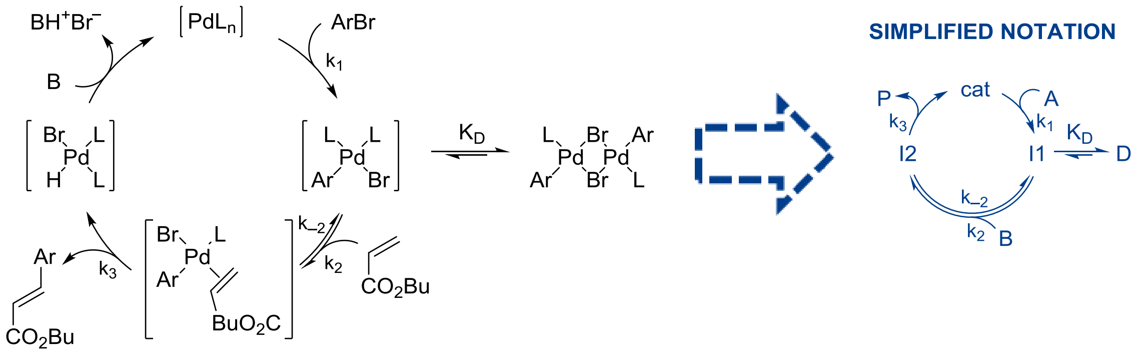

Steady-state approximation: $\frac{d[cat]}{dt} = k_3[I2] - k_1[A][cat] \approx 0$ (1)

Steady-state approximation: $\frac{d[I2]}{dt} = k_2[B][I1] - (k_3 + k_{-2})[I2] \approx 0$ (2)

Quasi-equilibrium assumption: $K_D = \frac{[D]}{[I1]^2}$ (3)

Mass Balance of catalytic species: $[Pd]_T = [cat] + [I1] + [I2] + 2[D]$ (4)

Rate law: $\frac{d[P]}{dt} = \frac{2k_3[Pd]_T}{b+\sqrt{b^2+4a[Pd]_T}}$ (5)

Where $a = 2K_D\left(\frac{k_3+k_{-2}}{k_2[B]}\right)^2$ and $b = \frac{k_2k_3[B]+k_1(k_3+k_{-2})[A]+k_1k_2[A][B]}{k_1k_2[A][B]}$ (6)

The normalized sensitivity is used as the equation for the order in catalyst (equation 7):

order of the reaction in [Pd]$_T$ $= \frac{\frac{dr}{d[Pd]_T}}{\frac{r}{[Pd]_T}} = \frac{b+\sqrt{b^2+4a[Pd]_T}}{2\sqrt{b^2+4a[Pd]_T}}$ (7)

The plot of equation 7 using the initial concentration values for the model is shown below:

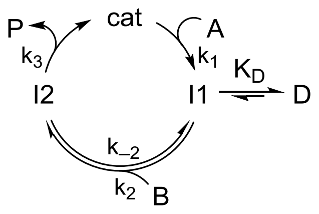

k_1 = 363 $M^{-1}h^{-1}$; k_2 = 34.2 $M^{-1}h^{-1}$; k_{-2} = 114 h^{-1}
k_3 = 374 h^{-1}; K_r = 9990 M^{-1}
$[A]_o$ = 0.20 M; $[B]_o$ = 0.28 M

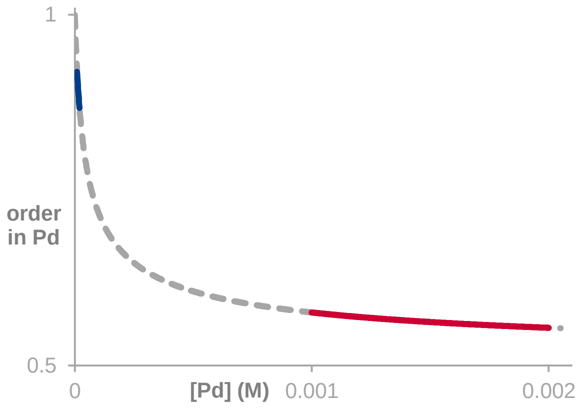

For the range of concentrations of catalyst analyzed in the manuscript (10^{-5} to $2\cdot10^{-5}$ M and 10^{-3} to $2\cdot10^{-3}$ M), the order in catalyst would vary during the course of the reaction. The average values of the order in catalyst at different stages of the reaction are shown below.

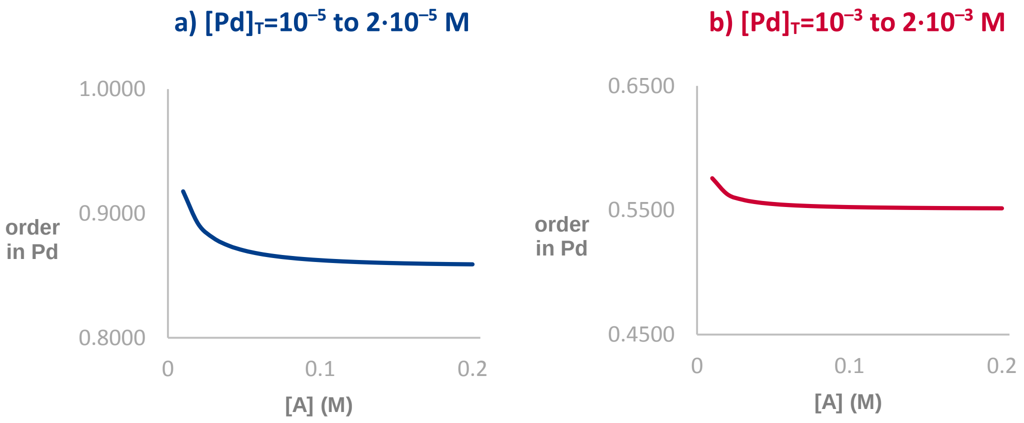

7. Figure 4b

The values of concentrations and kinetic constants used to simulate figure 3 are shown below.

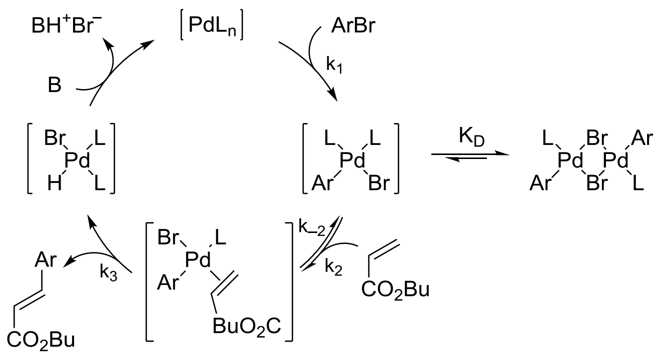

$[A]_o = 0.20$ M; $[B]_o = 0.28$ M

$k_1 = 363\ M^{-1}h^{-1}$; $k_2 = 34.2\ M^{-1}h^{-1}$; $k_{-2} = 114\ h^{-1}$; $k_3 = 374\ h^{-1}$; $K_D = 9990\ M^{-1}$

The simulated concentration results against time for the different concentrations of catalyst (10^{-5} M, $5{\cdot}10^{-3}$ mol%; $1.5{\cdot}10^{-5}$ M, $7.5{\cdot}10^{-3}$ mol%; $2{\cdot}10^{-5}$ M, 0.01 mol%; 10^{-3} M, 0.5 mol%; $1.5{\cdot}10^{-3}$ M, 0.75 mol%; $2{\cdot}10^{-3}$ M, 1 mol%) are in the section 7.5 of this supporting information.

8. Numerical data

8.1. Figure 2a

time (h)	[A] (M) ([cat]$_o$ = 0.05 M)	[A] (M) ([cat]$_o$ = 0.02 M)	[A] (M) ([cat]$_o$ = 0.01 M)	[A] (M) ([cat]$_o$ = 0.005 M)
0.00	1.013	1.003	1.015	1.001
0.03	0.847	0.929	0.981	0.979
0.06	0.681	0.866	0.922	0.968
0.08	0.586	0.809	0.895	0.928
0.11	0.476	0.752	0.863	0.935
0.14	0.379	0.703	0.850	0.940
0.17	0.320	0.649	0.812	0.909
0.19	0.251	0.603	0.788	0.892
0.22	0.201	0.557	0.742	0.858
0.25	0.146	0.509	0.737	0.842
0.28	0.124	0.483	0.711	0.824
0.31	0.102	0.438	0.670	0.839
0.33	0.084	0.391	0.659	0.812
0.36	0.056	0.374	0.621	0.799
0.39	0.036	0.324	0.599	0.787
0.42	0.037	0.316	0.580	0.771
0.44	0.038	0.301	0.569	0.765
0.47	0.016	0.273	0.540	0.724
0.50	0.026	0.231	0.527	0.715
0.53	0.003	0.229	0.496	0.721
0.56	0.034	0.204	0.483	0.701
0.58	0.004	0.172	0.447	0.679
0.61	0.003	0.169	0.437	0.683
0.64	0.031	0.159	0.417	0.681
0.67	0.007	0.144	0.416	0.657
0.69	0.009	0.122	0.379	0.650
0.72	0.002	0.112	0.372	0.612
0.75	-0.003	0.110	0.364	0.607
0.78	-0.012	0.108	0.345	0.596
0.81	0.003	0.092	0.337	0.620
0.83	-0.018	0.075	0.307	0.574
0.86	-0.005	0.079	0.326	0.559
0.89	-0.006	0.073	0.293	0.578
0.92	-0.017	0.047	0.283	0.553
0.94	0.002	0.053	0.270	0.534
0.97	-0.009	0.053	0.236	0.521
1.00	0.004	0.042	0.237	0.513
1.03	-0.008	0.057	0.246	0.512
1.06	0.026	0.041	0.218	0.493
1.08	-0.006	0.028	0.218	0.476
1.11	0.009	0.010	0.191	0.473
1.14	-0.014	0.033	0.194	0.457
1.17	0.006	0.017	0.188	0.454
1.19	0.010	0.026	0.173	0.459

1.22	0.014	0.015	0.175	0.443
1.25	0.014	0.013	0.160	0.431
1.28	0.002	0.020	0.148	0.406
1.31	0.004	0.021	0.150	0.418
1.33	0.000	0.016	0.146	0.410
1.36	-0.002	0.001	0.129	0.384
1.39	0.009	0.010	0.116	0.397
1.42	-0.010	0.006	0.107	0.378
1.44	0.007	0.026	0.117	0.369
1.47	0.012	0.019	0.097	0.369
1.50	0.006	0.005	0.099	0.365
1.53	-0.008	0.008	0.105	0.344
1.56	-0.012	0.002	0.099	0.359
1.58	-0.009	0.002	0.080	0.332
1.61	0.009	0.008	0.087	0.319
1.64	-0.008	0.010	0.079	0.323
1.67	0.001	-0.019	0.076	0.305
1.69	0.008	0.006	0.071	0.320
1.72	-0.003	0.015	0.053	0.290
1.75	0.006	0.022	0.071	0.287
1.78	-0.010	0.000	0.062	0.288
1.81	-0.010	0.008	0.068	0.285
1.83	-0.009	-0.013	0.057	0.264
1.86	-0.006	-0.001	0.060	0.267
1.89	0.005	-0.007	0.052	0.231
1.92	-0.008	-0.003	0.060	0.261
1.94	0.003	0.002	0.034	0.254
1.97	0.023	0.015	0.047	0.232
2.00	0.005	-0.005	0.046	0.243
2.03	-0.007	-0.004	0.048	0.226
2.06	-0.002	0.021	0.042	0.228
2.08	0.009	0.013	0.050	0.224
2.11	0.006	0.011	0.042	0.219
2.14	-0.007	-0.018	0.034	0.202
2.17	-0.003	-0.001	0.045	0.208
2.19	0.008	-0.008	0.051	0.197
2.22	-0.005	0.010	0.033	0.196
2.25	0.006	0.013	0.022	0.200
2.28	-0.007	-0.004	0.019	0.197
2.31	-0.009	0.010	0.028	0.199
2.33	-0.011	0.004	-0.001	0.182
2.36	-0.010	0.003	0.013	0.165
2.39	0.013	0.004	0.024	0.181
2.42	0.000	-0.003	0.023	0.169
2.44	0.000	-0.009	0.010	0.150
2.47	0.001	-0.012	0.011	0.164
2.50	-0.004	-0.005	0.046	0.154
2.53	-0.005	-0.003	0.007	0.157
2.56	-0.010	0.014	0.029	0.170
2.58	0.004	-0.006	0.031	0.145

2.61	-0.001	-0.002	0.007	0.167
2.64	0.002	0.001	0.024	0.145
2.67	0.002	-0.016	0.004	0.132
2.69	-0.010	0.014	0.032	0.131
2.72	0.009	-0.003	-0.004	0.127
2.75	0.014	0.008	0.013	0.124
2.78	0.003	0.006	0.005	0.124
2.81	0.010	0.001	0.000	0.112
2.83	-0.009	0.007	0.031	0.113
2.86	-0.009	-0.003	0.005	0.109
2.89	-0.003	-0.003	0.013	0.120
2.92	0.004	0.022	0.020	0.137
2.94	-0.022	0.017	-0.001	0.110
2.97	0.004	0.011	0.013	0.091
3.00	0.003	0.009	0.007	0.102
3.03	-0.006	-0.005	0.021	0.133
3.06	-0.020	0.010	0.007	0.110
3.08	-0.010	-0.005	-0.003	0.111
3.11	0.003	-0.002	0.012	0.084
3.14	0.015	0.015	-0.041	0.093
3.17	-0.011	-0.012	0.022	0.068
3.19	0.006	-0.016	0.009	0.087
3.22	-0.002	-0.012	0.005	0.085
3.25	-0.014	0.006	0.006	0.083
3.28	0.010	0.017	-0.003	0.090
3.31	-0.007	-0.017	-0.004	0.068
3.33	-0.006	-0.002	0.006	0.078
3.36	0.005	-0.017	-0.008	0.072
3.39	-0.018	-0.008	-0.005	0.079
3.42	0.001	0.008	-0.004	0.056
3.44	0.001	-0.004	-0.009	0.071
3.47	0.017	0.006	-0.010	0.088
3.50	0.003	0.005	0.006	0.056
3.53	0.000	-0.007	0.013	0.054
3.56	0.016	-0.013	-0.007	0.072
3.58	-0.014	0.005	0.009	0.053
3.61	0.005	-0.010	-0.011	0.050
3.64	0.006	-0.019	0.006	0.054
3.67	-0.001	0.005	-0.006	0.051
3.69	0.006	0.008	0.004	0.056
3.72	-0.003	-0.008	0.008	0.047
3.75	0.001	0.000	-0.002	0.064
3.78	-0.006	-0.035	-0.005	0.049
3.81	-0.005	-0.004	-0.008	0.056
3.83	0.022	0.012	0.006	0.047
3.86	-0.008	0.002	0.000	0.048
3.89	0.019	0.016	-0.003	0.055
3.92	-0.010	0.000	0.012	0.061
3.94	0.005	0.003	-0.008	0.047
3.97	0.029	-0.003	0.002	0.037

4.00	-0.012	-0.003	-0.011	0.046
4.03	-0.011	0.007	0.001	0.040
4.06	0.012	0.005	-0.003	0.044
4.08	0.000	0.011	-0.002	0.057
4.11	-0.025	-0.011	0.014	0.039
4.14	-0.017	-0.004	0.001	0.048
4.17	-0.008	0.006	-0.005	0.044
4.19	0.003	-0.007	0.015	0.025
4.22	-0.006	0.001	0.002	0.040
4.25	0.000	-0.017	0.000	0.018
4.28	0.000	-0.017	-0.011	0.036
4.31	-0.002	-0.006	-0.009	0.034
4.33	0.003	-0.002	-0.001	0.056
4.36	0.021	-0.002	-0.013	0.048
4.39	-0.002	0.001	0.003	0.034
4.42	-0.008	-0.004	-0.010	0.019
4.44	-0.005	-0.007	0.015	0.028
4.47	0.000	-0.005	-0.013	0.034
4.50	0.001	-0.005	0.013	0.036
4.53	0.002	-0.009	0.010	0.004
4.56	0.001	0.025	-0.005	0.028
4.58	0.006	0.013	-0.005	0.008
4.61	0.009	-0.015	-0.009	0.018
4.64	-0.001	0.010	0.021	0.014
4.67	-0.008	-0.001	0.000	0.022
4.69	-0.001	0.006	0.011	0.028
4.72	-0.018	0.000	-0.002	0.022
4.75	-0.014	0.003	0.003	0.034
4.78	0.003	0.005	0.010	0.021
4.81	0.018	-0.011	-0.004	0.030
4.83	0.008	-0.017	0.000	0.016
4.86	-0.003	0.000	-0.007	0.024
4.89	0.008	-0.008	-0.007	0.011
4.92	-0.008	-0.016	0.014	0.019
4.94	0.011	0.006	0.008	0.020
4.97	-0.010	-0.015	-0.002	0.028
5.00	-0.006	0.003	-0.009	0.013
5.03	0.003	0.006	0.004	0.029
5.06	0.004	0.001	0.011	0.006
5.08	-0.009	0.007	0.008	0.016
5.11	-0.003	0.003	-0.008	0.008
5.14	-0.008	0.012	0.010	0.005
5.17	-0.001	0.000	-0.010	0.011
5.19	0.005	-0.006	-0.017	0.029
5.22	0.000	-0.013	-0.018	0.024
5.25	0.011	-0.010	0.006	0.024
5.28	0.006	0.000	0.018	0.043
5.31	-0.008	0.005	-0.016	0.013
5.33	-0.001	0.010	-0.010	0.011
5.36	0.004	0.011	-0.002	0.010

5.39	-0.002	-0.014	0.000	-0.008
5.42	0.004	0.002	0.013	0.008
5.44	-0.009	-0.008	0.011	0.024
5.47	0.004	-0.001	-0.008	0.000
5.50	-0.009	-0.001	0.007	0.013
5.53	0.009	0.000	-0.017	0.013
5.56	-0.012	-0.003	0.005	-0.007
5.58	-0.017	-0.005	0.017	0.018
5.61	0.003	-0.011	-0.007	0.026
5.64	0.004	-0.003	0.020	0.005
5.67	0.000	-0.010	0.003	0.012
5.69	-0.008	0.000	-0.003	0.020
5.72	-0.013	0.004	-0.002	0.006
5.75	-0.008	-0.006	-0.004	0.007
5.78	0.023	-0.015	-0.002	0.004
5.81	-0.005	-0.004	0.017	0.005
5.83	0.003	-0.023	-0.012	0.021
5.86	-0.021	0.007	0.014	0.023
5.89	0.009	0.008	-0.001	0.009
5.92	0.006	0.009	0.002	-0.003
5.94	0.005	-0.007	-0.005	0.033
5.97	0.021	-0.005	0.024	0.025
6.00	-0.006	-0.002	0.001	-0.001
6.03	0.004	-0.013	0.011	0.004
6.06	-0.003	0.017	0.005	0.002
6.08	-0.012	-0.005	0.015	0.002
6.11	0.021	-0.001	-0.004	0.002
6.14	-0.001	0.008	-0.012	0.022
6.17	0.011	0.003	-0.001	0.019
6.19	0.002	-0.012	0.002	0.005
6.22	-0.014	-0.002	-0.005	0.012
6.25	0.000	0.002	-0.002	-0.012
6.28	0.008	-0.031	0.004	0.012
6.31	0.009	-0.010	0.001	-0.002
6.33	0.018	-0.008	-0.014	0.013
6.36	0.002	0.002	0.005	0.007
6.39	0.008	-0.004	0.010	0.021
6.42	0.002	0.012	-0.004	0.008
6.44	-0.012	0.014	-0.010	0.000
6.47	-0.008	0.001	0.003	-0.017
6.50	0.006	-0.006	-0.008	0.012
6.53	0.017	-0.006	-0.002	0.013
6.56	-0.007	0.011	-0.008	0.025
6.58	-0.012	-0.013	0.005	0.029
6.61	-0.001	0.001	0.003	-0.001
6.64	0.010	0.000	-0.002	0.018
6.67	0.004	-0.006	0.011	-0.010
6.69	0.006	0.004	0.008	0.011
6.72	0.012	0.005	-0.015	-0.009
6.75	0.005	-0.016	0.006	0.008

6.78	-0.007	0.000	0.001	-0.004
6.81	-0.012	-0.018	-0.002	0.001
6.83	0.016	0.001	0.007	-0.011
6.86	0.008	-0.007	0.020	-0.004
6.89	0.005	0.009	-0.003	0.014
6.92	0.000	0.003	0.011	-0.006
6.94	0.005	-0.009	0.008	-0.001

8.2. Figure 2b

time (h)	[A] (M) ($[cat]_o$ = 0.05 M)	time (h)	[A] (M) ($[cat]_o$ = 0.005 M)
0.00	0.986	0.00	1.001
0.03	0.847	0.69	0.650
0.11	0.476	1.64	0.323
0.22	0.201	2.94	0.110
0.36	0.056	4.28	0.036

8.3. Figure 2c

time (h)	[A] (M) ($[cat]_o$ = 0.05 M)	[A] (M) ($[cat]_o$ = 0.02 M)	[A] (M) ($[cat]_o$ = 0.01 M)	[A] (M) ($[cat]_o$ = 0.005 M)
0.00	1.037	1.099	1.017	1.057
0.03	0.802	0.969	1.004	1.103
0.06	0.678	0.837	0.944	0.979
0.08	0.648	0.868	0.897	0.889
0.11	0.500	0.712	0.896	0.979
0.14	0.382	0.762	0.827	0.936
0.17	0.224	0.724	0.843	0.853
0.19	0.259	0.611	0.834	0.781
0.22	0.244	0.520	0.709	0.907
0.25	0.129	0.570	0.674	0.870
0.28	0.170	0.446	0.754	0.860
0.31	0.140	0.434	0.633	0.725
0.33	0.127	0.427	0.659	0.803
0.36	0.019	0.372	0.626	0.811
0.39	0.049	0.429	0.622	0.804
0.42	0.018	0.244	0.566	0.786
0.44	-0.061	0.224	0.633	0.779
0.47	-0.055	0.272	0.466	0.718
0.50	0.102	0.125	0.562	0.714
0.53	-0.021	0.185	0.507	0.726
0.56	0.011	0.243	0.512	0.693
0.58	-0.023	0.161	0.321	0.751
0.61	-0.029	0.228	0.412	0.734
0.64	0.013	0.133	0.457	0.640
0.67	-0.026	0.141	0.442	0.667
0.69	0.003	0.182	0.417	0.608

0.72	0.061	0.049	0.346	0.582
0.75	-0.038	0.076	0.347	0.641
0.78	-0.038	0.073	0.356	0.497
0.81	0.055	0.084	0.325	0.622
0.83	-0.017	-0.016	0.285	0.565
0.86	-0.099	0.142	0.390	0.553
0.89	0.029	0.156	0.358	0.548
0.92	0.022	-0.002	0.369	0.588
0.94	-0.028	0.056	0.224	0.522
0.97	0.040	-0.026	0.211	0.567
1.00	0.028	0.080	0.158	0.522
1.03	0.013	-0.017	0.270	0.536
1.06	0.074	0.121	0.264	0.423
1.08	0.063	0.053	0.190	0.427
1.11	0.010	0.024	0.211	0.466
1.14	0.091	0.035	0.232	0.480
1.17	0.000	0.064	0.251	0.478
1.19	-0.035	0.023	0.048	0.472
1.22	-0.012	-0.028	0.150	0.550
1.25	-0.065	0.084	0.098	0.362
1.28	0.087	-0.002	0.152	0.454
1.31	0.064	-0.022	0.124	0.392
1.33	-0.095	-0.047	0.142	0.419
1.36	-0.018	0.023	0.031	0.393
1.39	0.098	0.071	0.133	0.293
1.42	-0.057	-0.017	0.042	0.377
1.44	0.045	-0.003	0.128	0.356
1.47	-0.029	0.017	0.143	0.421
1.50	0.053	-0.008	0.114	0.341
1.53	-0.032	0.014	0.166	0.274
1.56	0.017	-0.066	0.049	0.283
1.58	0.016	0.033	0.086	0.355
1.61	-0.003	0.009	0.129	0.245
1.64	-0.078	0.054	0.107	0.298
1.67	0.049	-0.008	0.020	0.375
1.69	0.081	-0.009	0.011	0.423
1.72	0.035	0.081	0.078	0.323
1.75	0.065	0.065	0.031	0.329
1.78	-0.018	0.037	-0.016	0.298
1.81	-0.042	0.069	0.040	0.371
1.83	0.078	-0.018	0.054	0.286
1.86	-0.044	0.058	0.008	0.257
1.89	-0.014	0.028	0.134	0.308
1.92	0.066	-0.013	-0.020	0.312
1.94	-0.024	0.021	0.106	0.201
1.97	-0.013	0.061	0.057	0.246
2.00	0.115	-0.003	0.056	0.241
2.03	0.008	0.050	0.023	0.244
2.06	-0.049	-0.076	0.115	0.129
2.08	-0.006	0.007	0.021	0.138

2.11	0.071	0.011	0.017	0.256
2.14	-0.013	-0.020	0.064	0.218
2.17	0.023	0.009	-0.007	0.216
2.19	0.058	0.032	-0.001	0.184
2.22	0.057	0.006	-0.001	0.272
2.25	-0.047	0.076	0.052	0.219
2.28	-0.109	-0.074	-0.012	0.205
2.31	-0.013	0.044	0.035	0.248
2.33	0.002	-0.022	0.070	0.097
2.36	0.004	0.060	0.076	0.195
2.39	0.047	0.060	0.077	0.110
2.42	-0.012	-0.022	-0.008	0.240
2.44	0.023	0.024	0.013	0.188
2.47	0.018	-0.136	0.052	0.199
2.50	-0.025	0.026	0.089	0.180
2.53	0.022	0.000	0.039	0.145
2.56	0.047	0.021	0.045	0.087
2.58	0.053	-0.020	0.097	0.084
2.61	0.036	-0.033	0.054	0.123
2.64	-0.066	-0.025	-0.050	0.215
2.67	0.029	0.104	0.052	0.077
2.69	-0.022	-0.040	-0.018	0.241
2.72	0.074	-0.052	-0.029	0.084
2.75	0.012	-0.017	0.004	0.117
2.78	0.050	0.032	0.073	0.070
2.81	0.038	0.039	-0.009	0.205
2.83	-0.003	0.018	-0.064	0.138
2.86	-0.008	-0.110	0.031	0.175
2.89	-0.002	0.051	-0.014	0.123
2.92	-0.030	0.057	-0.003	0.074
2.94	-0.020	0.012	-0.016	0.152
2.97	-0.013	0.001	0.098	0.132
3.00	0.004	0.075	-0.037	0.085
3.03	0.050	0.073	0.083	0.091
3.06	0.019	-0.047	-0.004	0.168
3.08	0.029	-0.032	-0.089	0.187
3.11	0.020	0.036	0.009	0.117
3.14	-0.043	0.048	-0.032	0.148
3.17	-0.045	0.107	0.000	-0.021
3.19	-0.002	-0.020	-0.046	0.100
3.22	0.091	0.061	-0.009	0.077
3.25	0.007	-0.017	0.007	0.074
3.28	-0.031	0.006	-0.029	0.067
3.31	-0.086	0.036	0.027	0.088
3.33	-0.041	-0.061	0.016	0.097
3.36	-0.040	-0.053	0.101	-0.038
3.39	0.034	0.040	0.031	0.027
3.42	0.003	0.016	0.039	0.010
3.44	0.001	0.060	-0.073	0.089
3.47	0.018	0.060	-0.028	0.051

3.50	0.028	0.065	0.018	-0.022
3.53	0.060	-0.020	-0.033	0.056
3.56	0.000	0.044	0.006	0.014
3.58	0.038	-0.036	-0.031	0.022
3.61	-0.010	0.034	0.035	0.073
3.64	0.008	0.068	-0.027	0.022
3.67	0.053	0.053	-0.016	0.090
3.69	0.020	0.099	0.050	0.022
3.72	-0.015	-0.114	-0.012	0.078
3.75	-0.035	-0.027	-0.003	0.130
3.78	0.012	-0.037	0.050	0.104
3.81	-0.037	-0.003	0.026	0.049
3.83	-0.056	-0.078	0.107	0.005
3.86	0.057	0.018	0.028	-0.007
3.89	-0.049	0.004	-0.110	0.077
3.92	-0.030	-0.031	0.054	0.107
3.94	0.045	0.024	0.048	0.052
3.97	-0.008	0.047	0.039	-0.035
4.00	0.031	0.095	0.103	0.053
4.03	0.017	0.014	0.062	-0.045
4.06	-0.018	-0.084	-0.043	0.092
4.08	0.034	-0.009	0.069	0.079
4.11	0.018	-0.018	-0.009	0.016
4.14	-0.038	-0.068	0.084	0.090
4.17	0.022	0.021	0.027	0.091
4.19	0.020	0.119	-0.081	-0.050
4.22	0.038	0.053	-0.011	-0.074
4.25	0.003	0.132	0.018	0.039
4.28	-0.010	0.036	-0.012	0.106
4.31	0.011	0.062	0.051	0.094
4.33	0.004	0.035	0.007	0.014
4.36	-0.033	-0.014	0.068	-0.020
4.39	0.013	-0.004	0.035	0.154
4.42	0.053	0.030	-0.085	-0.013
4.44	0.038	0.048	-0.009	0.039
4.47	-0.072	-0.012	-0.021	0.013
4.50	0.010	0.043	0.024	0.085
4.53	-0.014	-0.019	0.104	-0.033
4.56	-0.001	-0.007	0.070	0.003
4.58	0.075	0.028	-0.026	-0.005
4.61	-0.037	0.075	0.015	-0.003
4.64	-0.074	0.016	-0.022	-0.019
4.67	-0.005	0.018	-0.042	-0.029
4.69	0.009	0.060	0.070	0.001
4.72	-0.029	0.081	0.098	0.094
4.75	0.007	0.134	0.058	0.039
4.78	-0.074	0.064	0.014	0.002
4.81	0.022	0.020	-0.020	0.014
4.83	-0.003	0.033	0.024	0.061
4.86	-0.071	0.063	0.047	0.012

4.89	-0.029	0.010	-0.008	-0.069
4.92	0.034	0.008	-0.022	0.018
4.94	-0.027	0.025	0.050	0.076
4.97	0.026	0.035	-0.018	-0.018
5.00	0.098	-0.044	0.041	0.010
5.03	-0.007	0.042	0.008	0.027
5.06	-0.077	-0.011	0.071	0.001
5.08	-0.138	-0.012	-0.007	0.145
5.11	0.000	0.028	0.084	-0.012
5.14	-0.020	0.044	0.026	0.052
5.17	0.047	0.057	-0.021	0.111
5.19	0.034	0.026	-0.025	0.045
5.22	-0.072	-0.010	0.053	0.071
5.25	0.125	-0.065	0.035	0.074
5.28	-0.010	-0.007	-0.114	0.019
5.31	-0.066	-0.021	0.073	-0.046
5.33	-0.094	-0.075	0.063	0.060
5.36	-0.019	-0.039	0.044	0.074
5.39	-0.030	-0.049	-0.042	0.016
5.42	0.154	0.088	-0.037	-0.023
5.44	-0.059	0.057	0.071	0.031
5.47	-0.006	0.006	0.024	-0.058
5.50	0.088	-0.008	-0.099	0.023
5.53	0.025	-0.014	-0.049	-0.078
5.56	0.007	0.021	-0.058	-0.051
5.58	-0.008	-0.004	-0.058	0.066
5.61	-0.030	-0.020	-0.025	-0.050
5.64	-0.114	0.014	-0.042	-0.091
5.67	-0.039	-0.075	0.040	0.053
5.69	0.065	0.045	-0.039	-0.074
5.72	0.026	-0.089	-0.058	0.033
5.75	0.070	-0.013	-0.055	-0.070
5.78	0.024	-0.007	-0.044	0.040
5.81	-0.067	0.038	0.064	-0.074
5.83	-0.051	-0.060	-0.142	-0.003
5.86	-0.008	-0.011	-0.004	0.009
5.89	0.021	-0.049	-0.025	0.026
5.92	0.036	0.015	-0.031	0.042
5.94	0.076	0.064	-0.047	-0.089
5.97	0.097	0.047	-0.019	0.093
6.00	0.094	0.047	-0.083	0.019
6.03	0.038	-0.041	-0.025	0.022
6.06	0.038	0.035	-0.007	-0.018
6.08	-0.045	0.008	-0.003	-0.003
6.11	-0.016	-0.029	0.011	0.029
6.14	0.018	-0.098	-0.063	0.109
6.17	0.024	0.043	0.152	0.022
6.19	0.014	-0.024	-0.014	-0.063
6.22	0.024	-0.131	-0.023	0.016
6.25	-0.018	-0.021	0.063	0.027

6.28	0.017	-0.081	-0.082	0.019
6.31	-0.024	-0.004	0.018	0.059
6.33	0.016	0.004	-0.013	0.015
6.36	0.007	0.084	-0.012	0.040
6.39	-0.034	-0.102	0.057	0.036
6.42	0.008	0.000	0.034	-0.083
6.44	-0.027	0.050	-0.075	0.025
6.47	0.041	-0.005	0.032	-0.012
6.50	0.001	0.102	0.071	-0.024
6.53	-0.137	0.015	-0.005	-0.058
6.56	-0.030	-0.012	0.040	-0.002
6.58	0.086	0.056	0.059	-0.065
6.61	0.078	-0.048	-0.047	-0.007
6.64	0.086	0.051	-0.048	-0.012
6.67	0.002	-0.004	0.073	0.039
6.69	-0.062	-0.014	0.011	-0.014
6.72	-0.105	-0.021	-0.004	-0.018
6.75	0.026	0.043	0.000	0.011
6.78	0.055	0.019	0.117	-0.027
6.81	0.038	-0.064	0.102	-0.054
6.83	-0.004	-0.003	-0.027	0.006
6.86	-0.087	0.036	0.126	0.012
6.89	0.122	-0.028	-0.078	0.009
6.92	0.043	-0.013	-0.009	0.082
6.94	-0.033	-0.056	0.019	-0.054

8.4. Figure 3

time (h)	[A] (M) ([cat]$_o$ = 0.0308 M)	[A] (M) ([cat]$_o$ = 0.02185 M)	[A] (M) ([cat]$_o$ = 0.0155 M)
0.00	3.625	3.625	3.625
0.56	1.509	2.381	2.950
1.11	0.554	1.500	2.375
1.67	0.193	0.915	1.891
2.22	0.066	0.546	1.491
2.78	0.022	0.322	1.166
3.33	0.008	0.189	0.906
3.89	0.003	0.110	0.700
4.44	0.001	0.064	0.539
5.00	0.000	0.037	0.413
5.56	0.000	0.022	0.316
6.11	0.000	0.013	0.242
6.67	0.000	0.007	0.184
7.22	0.000	0.004	0.141
7.78	0.000	0.002	0.107
8.33	0.000	0.001	0.081
8.89	0.000	0.001	0.062
9.44	0.000	0.000	0.047
10.00	0.000	0.000	0.036

10.56	0.000	0.000	0.027
11.11	0.000	0.000	0.021
11.67	0.000	0.000	0.016
12.22	0.000	0.000	0.012
12.78	0.000	0.000	0.009
13.33	0.000	0.000	0.007
13.89	0.000	0.000	0.005

8.5. Figure 4b

time (h)	[A] (M) ($[cat]_o = 2·10^{-5}$ M)	[A] (M) ($[cat]_o = 1.5·10^{-5}$ M)	[A] (M) ($[cat]_o = 10^{-5}$ M)
0	0.200	0.200	0.200
175	0.165	0.171	0.179
350	0.134	0.146	0.159
525	0.107	0.123	0.141
700	0.084	0.102	0.125
875	0.064	0.084	0.109
1050	0.046	0.067	0.095
1225	0.031	0.052	0.082
1400	0.018	0.039	0.070
1575	0.008	0.028	0.059
1750	0.001	0.018	0.049
1925	0.000	0.009	0.040
2100	0.000	0.003	0.031
2275	0.000	0.001	0.024
2450	0.000	0.000	0.017
2625	0.000	0.000	0.011
2800	0.000	0.000	0.006
2975	0.000	0.000	0.002
3150	0.000	0.000	0.001
3325	0.000	0.000	0.000
3500	0.000	0.000	0.000

time (h)	[A] (M) ($[cat]_o = 2·10^{-3}$ M)	[A] (M) ($[cat]_o = 1.5·10^{-3}$ M)	[A] (M) ($[cat]_o = 10^{-3}$ M)
0	0.200	0.200	0.200
6	0.186	0.188	0.190
18	0.162	0.168	0.174
30	0.141	0.149	0.159
42	0.122	0.132	0.144
54	0.104	0.116	0.131
66	0.088	0.101	0.118
78	0.073	0.087	0.106
90	0.060	0.075	0.095
102	0.047	0.063	0.084
114	0.036	0.052	0.074
126	0.026	0.043	0.065

138	0.017	0.033	0.056
150	0.009	0.025	0.048
162	0.002	0.017	0.040
174	0.000	0.010	0.033
186	0.000	0.004	0.027
198	0.000	0.000	0.020
210	0.000	0.000	0.014
222	0.000	0.000	0.009
234	0.000	0.000	0.004
246	0.000	0.000	0.001
258	0.000	0.000	0.000
270	0.000	0.000	0.000
282	0.000	0.000	0.000
300	0.000	0.000	0.000
